# Antarctic Snow Failure Mechanics: Analysis, Simulations, and Applications

**DOI:** 10.3390/ma17071490

**Published:** 2024-03-25

**Authors:** Enzhao Xiao, Shengquan Li, Ali Matin Nazar, Ronghua Zhu, Yihe Wang

**Affiliations:** 1 Polar Research Institute of China, Shanghai 200136, China; xiaoenzhao@pric.org.cn; 2 Ocean College, Zhejiang University, Zhoushan 316021, China; shengquan.li@zju.edu.cn; 3 Zhejiang University-University of Illinois at Urbana-Champaign Institute, Zhejiang University, Haining 314400, China; matin.22@intl.zju.edu.cn

**Keywords:** Antarctic snow, failure analysis, theoretical modeling, numerical methods, Antarctic infrastructures

## Abstract

Snow failure is the process by which the stability of snow or snow-covered slopes is destroyed, resulting in the collapse or release of snow. Heavy snowfall, low temperatures, and volatile weather typically cause consequences in Antarctica, which can occur at different scales, from small, localized collapses to massive avalanches, and result in significant risk to human activities and infrastructures. Understanding snow damage is critical to assessing potential hazards associated with snow-covered terrain and implementing effective risk mitigation strategies. This review discusses the theoretical models and numerical simulation methods commonly used in Antarctic snow failure research. We focus on the various theoretical models proposed in the literature, including the fiber bundle model (FBM), discrete element model (DEM), cellular automata (CA) model, and continuous cavity-expansion penetration (CCEP) model. In addition, we overview some methods to acquire the three-dimensional solid models and the related advantages and disadvantages. Then, we discuss some critical numerical techniques used to simulate the snow failure process, such as the finite element method (FEM) and three-dimensional (3D) material point method (MPM), highlighting their features in capturing the complex behavior of snow failure. Eventually, different case studies and the experimental validation of these models and simulation methods in the context of Antarctic snow failure are presented, as well as the application of snow failure research to facility construction. This review provides a comprehensive analysis of snow properties, essential numerical simulation methods, and related applications to enhance our understanding of Antarctic snow failure, which offer valuable resources for designing and managing potential infrastructure in Antarctica.

## 1. Introduction

Scientific research in the Antarctic region faces various challenges due to snow and ice covering 98% of the land all year round, along with frequent storms. These difficulties include the threat of avalanches, snowstorms that erode buildings, and the construction and maintenance of snow runways [1]. In the study of snow, it is necessary to consider that snow is distinguished from ice by its porous structure, containing purified water, and particle properties depending on the atmospheric conditions at the formation [2]. By studying the mechanical properties of snow blocks, such as density, porosity, crystal structure [3], and sintering properties [4], including the internal and external factors mainly affected by humidity, temperature, metamorphism, and wind erosion, we could better understand the structural change process of snow blocks through the mechanics experiment of snow failure.

However, we found that more than experiments are needed to accurately and efficiently observe the change process of snow microstructure. Due to the complex structure of snow and its sensitivity to environmental conditions, systematic experimental exploration becomes difficult, making it challenging to observe the changes in snow at the microscopic level [5]. In general, considering the elastoplastic properties of snow, the Mohr–Coulomb criterion [6] is adopted as the analytical theory of snow failure, and the theoretical criterion is updated with the progress of research [7]. In addition, there still needs to be a theoretical model that accurately describes the process of snow failure. Therefore, numerical simulation methods have been used to simulate and verify the experimental process and experimental results and evaluate different experimental parameters.

For more than a decade, researchers have used different methods to obtain 3D microstructures of snow blocks, such as serial sections in snow specimens [8] and X-ray computed microtomography [9], and have combined them with models such as open-cell foam [10] and fiber bundles [11] to more accurately reveal the internal structure of snow. Subsequently, using finite element and discrete element methods to simulate the internal changes during snow failure in numerical studies can provide more accurate predictions for practical experiments of Antarctic snow blocks. Furthermore, although many studies have examined large-scale snow failure mechanisms, some accurate analysis models for the snow flow and failure conduction process in a single or specific avalanche still need to be included. By employing numerical simulation methods, researchers can gain a deeper understanding of the mechanisms and impacts of snow damage at different scales, such as the fracture conduction of snow layers during avalanche events, and can use an appropriate method to prevent avalanches and minimize snow failure damage.

The purpose of this current review is to enhance our comprehension of the characteristics of Antarctic snow by investigating the mechanical experiment and numerical simulation methods of snow failure to more precisely analyze the mechanism behind it, so as to provide more convenience for future polar engineering construction. First, we reviewed the main material properties of snow and the important factors that exert a profound influence on it and outlined the mechanical failure experiments of snow blocks such as compression, shear, and mixing mode, so as to acquire a deeper comprehension of the structural change situations during the failure of snow blocks. Secondly, in order to delve into the micro-level phenomena associated with snow block failure, researchers incorporated some numerical simulation methods. Here, we first reviewed several approaches to obtain a three-dimensional model of a snow block. Subsequently, some analysis models in snow block failure from the micro to the macro level were elucidated, thereby enabling researchers to analyze the intricate mechanisms behind snow crystal interactions within the snow block. Finally, some conclusions were put forward, which consider the advantages and disadvantages of analysis models used in snow failure, as well as provide the theoretical basis for predicting and preventing avalanches and constructing and protecting the snow runways.

## 2. Failure Types and Mechanical Characterization of Antarctic Snow

Snow is formed by the polymerization of ice crystals, but unlike ice, there are many pores inside, and the structure is very complex. The formation of snow is affected by temperature, humidity, wind, and atmospheric conditions and changes rapidly and continuously [12], which affects the properties of the snow itself. Here, we will divide the discussion into two parts; one is the natural properties of snow, such as snow density and the snow particle size range. Moreover, the discussion will be on factors that affect its nature, such as snow thickness and the impact of seasonal changes, which help us understand the snow itself intuitively. The second part of the discussion will describe some related experiments in order to understand the process and significance of snow failure and deformation in practical experiments.

### 2.1. Antarctic Snow Characteristics and Temperature Effect

#### 2.1.1. Snow Density

As the most widely studied physical characteristic of snow, density can directly distinguish the difference between snow blocks. Snow density measurement methods in Antarctica include X-ray micro-focus computed tomography, neutron density, gamma ray attenuation, and optical televiewer (OPTV) [13]. 

Generally speaking, the density of fresh snow ranges from 10 to 600 kg/m^3^, while the density above 600 kg/m^3^ is snow that has been subjected to wind wear and multiple thawing cycles [14]. In the marine environment, the density of fresh snow is usually between 100 and 300 kg/m^3^. However, in continental climates, fresh snow’s density is generally between 10 and 100 kg/m^3^ [15]. According to the data that we examined for this study, we found that the density of snow varies seasonally [16,17]. Fons et al. (2023) divided the mean snow density of the southeast polar region of Antarctica into 360 (summer), 350 (autumn), 330 (winter), and 310 (spring) kg/m^3^, respectively, showing that the snow density varies slightly with seasonal changes [18]. These seasonal values were linearly interpolated between the midpoint dates, which provided estimates of the daily snow density for use in a snow thickness calculation. The seasonal variation in snow density is due to several physical processes after new snow begins to deposit, including the weight compaction of new snow layers, melt-refreezing, dry and wet snow mass metamorphism, and wind erosion. Among them, the seasonal snow cover structure results from the continuous overlaying of snow layers, usually divided into a high-density layer at the top and a thick, low-density dark gray layer at the bottom [19]. Yanchukovsky et al. (2021) also concluded that the density of snow cover depends on its depth by using neutron flux monitoring [20]. Meltwater refreezing contributes to the apparent difference in density within the snowpack. Usually, in summer, meltwater may be produced in coastal areas of East Antarctica and on the surface of ice shelves [21] and freeze at some depth within the cold snowpack. However, the specific effects of the meltwater refreezing process on the density stratification within the snowpack and its relationship with the surface mass balance of snow blocks remain unclear [22]. In seasonal snow cover, the humidity ratio in accumulated snow makes the snow density have prominent nonlinear behavior characteristics. De Michele et al. (2013) applied a one-dimensional model for the temporal dynamics of the snowpack, classifying snow cover into a mixture of two components, a dry part, including ice structure and air, and a wet part consisting of liquid water, emphasizing the importance of considering liquid water in dynamic experimental simulations of snow density [23]. 

In addition, wind erosion and metamorphism also significantly impact snow density. In the central plateau of the northern mountains of the Antarctic Peninsula, there is a dry snow zone, where years of wind erosion reshaped the outer snow crystals closer to a sphere shape and bound them more tightly together, resulting in a higher density of snow patches. Instead, the constructive metamorphism caused by the solid vertical temperature gradient of the dry snow layer results in larger faceted particles that cannot be packed tightly together, resulting in a low densification rate of the snow blocks [24,25]. Wiesmann et al. used twenty snow samples to conduct a section analysis [26]; Table 1 shows some properties of these snow samples.

Snow’s characteristics and physical change process are closely related to density, which is very important for the study of snow mass destruction. In addition, the characterizing of snow density is helpful in obtaining hydrological data such as the seasonal variation in snow cover and snowmelt runoff [27], which is very helpful for understanding the local snow environment.

#### 2.1.2. Snow Structural Characteristics

This section is divided into morphology and thermodynamics to explain the structural characteristics of snow. The former summarizes the classification of snow crystals, the range of snow particle size, and polar conditions. The latter outlines the thermodynamic classification of snow and its specific sintering phenomena.

**Figure 1:**
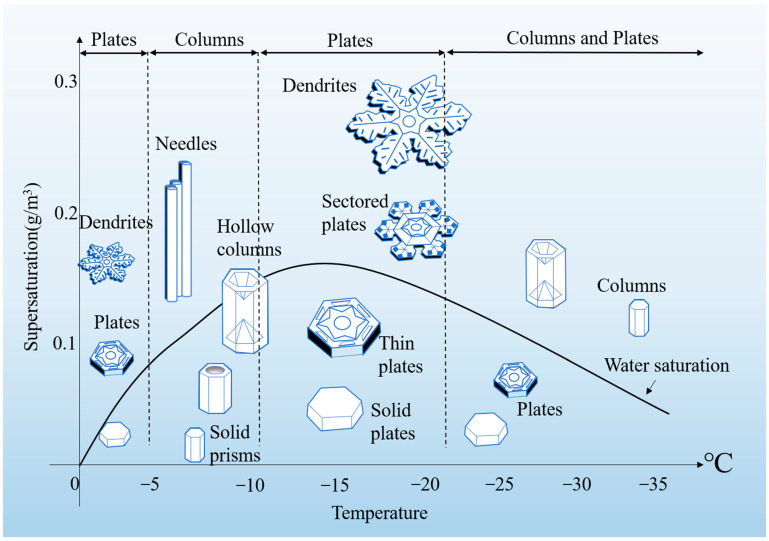
**A summary of snow crystal characteristics.** The Nayaka diagram illustrates which snow crystal forms appear at different temperatures and supersaturation [38].

Bonds between snow crystals and the growth of snow crystals are strongly influenced by temperature and humidity. As shown in Figure 1, supersaturation determines the exact shape of the snow crystals. When the supersaturation is low, the crystal formed is completely dense with a flat surface. As supersaturation increases, the crystal becomes more and more hollow; the formation of the dendritic structure is also observed under conditions of high supersaturation and rapid growth [28]. In terms of appearance, common snow crystal forms can be roughly divided into dendrite, fern dendrite, classic dendrite, simple star-shaped dendrite, needle, sandwich plate, and dendrite with broadening arms [29]. In general, snow crystals at colder temperatures also show a more symmetrical structure of polyhedral crystals. In the Antarctic low-temperature environment, typical snow crystal shapes are “Gohei twins” and “Spearhead”. One of the characteristics is the prismatic surface growth anomaly and symmetrical structure [30]. Some researchers have analyzed the changes in the neck region between adjacent snow particles, finding that bond growth and grain geometry strengthen the influence of the neck region [31]. Toyota et al. (2007) found that the most prominent features of snow particles were polyhedral crystals and deep chalk, which accounted for about 78% of the total snow depth, far greater than the proportion of spherical particles in the snow [32].

Further, in addition to the crystal form of snow, snow as an acceptable medium that is particularly active in thermodynamics can be divided into dry snow and wet snow according to its liquid water content and whether it is at melting temperature [33]. The snow crystals in dry snow mainly exist in two forms: one form is to affect the equilibrium form of the particle shape, which means the water vapor diffusion in dry snow is limited, and the growth rate of snow crystals is reduced to achieve the equilibrium state, and the proportion of circular grains is generally more obvious. The second form is the dynamic growth form, in which the proportion of polycrystalline grains is large. The growth rate of snow crystals in dry snow decreases with snow density but increases with the increase in the temperature gradient [34], because a large temperature gradient may lead to steam pressure and heat convection, which accelerate the growth rate of snow crystals [35]. Sturm et al. (1997) found that the thermal characteristics of snow usually change with the density [36]. Zhang et al. (1996) studied the influence of snow thickness on the ground thermal conductivity state by using a one-dimensional phase-change heat flow finite difference model. It is found that the deep snow layer is very important to the thermal insulation effect of seasonal snow [19]. Meanwhile, the metamorphism of dry snow can be divided into weak gradient metamorphism or temperature gradient metamorphism, and the weak gradient metamorphism process is similar to the process of snow sintering. Snow particles make contact with each other due to pressure to form a bond, providing solid cohesion. In the process of temperature gradient metamorphism, the temperature decreases layer by layer from the ground layer to the top layer, and the heat is transferred from the high-temperature region to the low-temperature region. At the same time, the water vapor in the melting or saturated state in the high-temperature region is also transferred to the low-temperature region, and the ice crystals are re-condensed. The local thermodynamic equilibrium changes also affect the types of ice crystals forming.

Wet snow can be divided into well-bonded snow and unbonded snow. Wet snow is tightly bonded when the liquid content is less than 7% of the overall volume. On the contrary, when the liquid content is high, the grain clusters and their significant grain boundaries are unstable, resulting in a decline in cohesiveness.

Regarding the size range of snow particles, generally speaking, the diameter of wind-blown snow particles is about 0.1 mm, and the crystal size of falling snow varies from about 0.1 mm to a few mm. However, fine particles proliferate after the snow is deposited, and branches of large dendritic crystals disappear, so dry surface snow generally tends to have an average (or median) particle size in the 0.4 to 1.0 mm range. There is little further grain growth in dry snow (unlike crystal growth), but wetting or thawing can produce grains a few millimeters in diameter, as does depth development and general temperature gradient metamorphism [35,37].

On the other hand, the sintering of snow also has a significant effect on the snow particles. For decades, there have been many studies on the sintering of ice [39,40] and the mechanical properties of ice sintering on different time scales ranging from months to sub-seconds [41]. However, sintering is also very important for snow, such as in analyzing the causes of avalanches [42,43] and buildings in polar regions [44]. In thermodynamics, the temperature of snow is always close to its melting point [45], and snow block sintering is the process of forming a bond between particles when the contact between snow particles is close to the melting point temperature. A single snow crystal is connected through sintering into a complex microstructure; the snow grain size and shape are also reshaped under a temperature gradient or pressure gradient. So, sintering is actually an early stage of the snow metamorphism process [45]. Hong et al. (2022) conducted an experimental study to observe the change in sintering density during the compression of snow blocks in the temperature range of −100 to 0 degrees and the pressure loading range of 0 to 100 MPa and found that pressure is the most prominent factor affecting sintering density [46]. While considering the increased rate of ice fabrication, low-temperature limits, and the reliable strength of sintered snow, high-pressure sintered snow is conducive to easy, widespread, and efficient utilization as a promising material for construction in polar regions [46].

By studying the shape of snow crystals, we can understand or obtain important information about the mechanical behavior and deformation mode of snow blocks because different shapes of crystals may have different stress distribution and deformation mechanisms. The study of the thermodynamic and sintering characteristics of snow blocks can also reveal the change and structural evolution of snow blocks, which help us understand the energy dissipation and stability change in the process of snow block failure.

### 2.2. Typical Failures and Mechanical Characterization Methods

Snow damage types are divided into four forms: shear failure, tensile failure, compression failure, and mixed failure. Its research content is also widely used in life, such as in the construction of snow tracks, house construction, sled boards, and snow tire contact friction in the Antarctic region.

#### 2.2.1. Snow Failure in Shear and Tension

The shear failure of snow blocks refers to the horizontal force that causes the internal structure to fail to maintain stability and thus to destroy and deform. Much of the literature has studied the destructive effects of shear friction on snow blocks under different internal and external conditions. De Montmollin et al. (1982) divided the shear failure effects of natural snow blocks into viscous and brittle according to different shear rates [47]. Using the Bevameter rotating device test, the viscous effect is obtained when the rotation rate is within 0.0075 rpm. Under the rotational shear force at low speed, the snow particles on the contact surface melt and bond when the rotational speed exceeds 0.0075 rpm. In the process of increasing the speed to 0.75 rpm, the brittle effect and even the cyclic effect of brittle failure are obtained, and the bonds between snow particles deteriorate rapidly, which means they break and regenerate after moving, but not all experimental process rates have been tested, so the measured stress levels are limited [47]. For the shear strength measurement of snow blocks, Nakamura et al. (2010) designed a vibration device that fixed snow blocks in the slide block and made the slide block vibrate back and forth along the Z-shaped track with the rotation of the motor [48]. The influence of the snow block density and overburden load on shear strength was discussed, which is very helpful for analyzing the cause of an avalanche and shear failure. For example, McClung et al. (1977) divided the shear strength of snow into cohesive force and friction force [6]. They tested the shear strength of snow blocks under different densities, different normal stresses, and loading rates within the range of 150 kg/m^3^ to 400 kg/m^3^ [6]. It was also observed that the samples with higher density or those undergoing rapid strain exhibited shorter snow block displacement at the time of failure. This means an avalanche could occur if the weak layer at the base of a slab were to be strain-softening. Meanwhile, Conway et al. (1984) suggested that the potential slippage of the snow failure during an avalanche was in the weak basement-layer region between the regions of high snow cover intensity [49].

In addition, it is necessary to consider the tensile fracture toughness of the upper end of the snow slab. Therefore, shear and tensile failures may exist simultaneously when an avalanche occurs [50]. Experimental studies show that the fracture toughness of snow under shear is roughly the same as that under tension. Although some studies have been conducted on the snow interface and shear strength, there are few studies on the shear behavior of the snow layer deposited on different rigid materials (e.g., concrete, polyvinyl chloride (PVC), and thermoplastic polyolefins (TPO)) [51]. Bartko et al. (2018) selected different roof materials and adjusted different angles [52]. They also observed the sliding friction of the snow sample and studied the distribution of the snow sample load [52]. Vallero et al. (2022) studied the shear strength of the interface between snow and mortar through two sets of temperatures (T1 = −10 °C and T2 = −5 °C) and two sets of sintering times (i.e., long sintering: LS = 20 h, short sintering: SS = 20 min) [53]. These two parameters were used to test their effects on interfacial shear strength. The friction behavior of the snow layer in contact with a rigid substrate (mortar block) was quantified [53].

Shear and tensile snow failure usually occur during avalanche events and in a mixed form. When a shear fracture in the weak layer or at the weak interface underlying the slab reaches the fracture threshold, the snow slab brittle fractures and loses its stability, leading to avalanche release [51]. In conclusion, it is difficult to simulate the mixed fracture in an experiment. It is not even easy to put the snow sample under pure traction or pure shear [54]. It is challenging to study the snow layer’s shear or tensile fracture on a large scale.

#### 2.2.2. Snow Failure in Compression

Snow is an ideal material with viscoelastic properties, which exhibits elastic, plastic, and brittle deformation effects during compression [55], depending on the compression rate and load. When slowly deformed, the snow mass undergoes a continuous deformation process and elastoplastic behavior, while rapid deformation results in an uninterrupted deformation process or brittle failure [56]. The studies show that the critical loading rate from plastic deformation to brittle deformation is between 10 mm/min and 30 mm/min at −25 °C and 0.6 g/cm^3^ density [55].

Meanwhile, the stress deformation curve and compressive strength of the snow block were thoroughly studied through a uniaxial compression experiment and a triaxial load compression experiment [57]. Scapozza et al. (2003) tested fine-grained and dry snow with a density range of 190–435 kg/m^3^ in a triaxial compression experiment and found that the relationship between yield stress and viscous strain rate is best described by a power law, similar to polycrystalline ice [58]. Lintzen et al. (2015) tested the triaxial compressive strength of machine-made snow of different structures [59]. They found that the compressive strength of snow blocks raises with increasing density and that old, coarser snow is significantly more brittle than new, fine-grained snow. In addition, changes in snow cover intensity are strongly influenced by factors such as the degree of binding between snow particles, temperature, and crystal size [60]. Sundu et al. (2024) repeatedly compressed snow blocks through load relaxation cycles, considering different types and relative densities of snow samples, as well as changes in geometric grain size, and revealed for the first time that the apparent change in the stress index in snow is driven by (geometric) grain size [61]. Ishiguro (2017) considered the influence caused by different contact surfaces when the snow block was compressed by loads and sequentially selected cylindrical, square, and rectangular cross-sectional pressure vessels to carry out the snow block compression experiment [62]. Another study has explored the effects of density between 270 and 340 kg/m^3^, the non-destructive uniaxial compression of fine snow, and grain-bond fracture regeneration on snow blocks’ deformation extent and elastoplastic variation [63].

These results have been widely used in laboratory and practical applications, especially for compacted snow runways at Antarctic airports [64]. The density and durability of the snow layer can be improved by breaking it down and sintering it or adding other materials, such as wood chips, to make it more solid and durable. Since Antarctic snow mainly consisted of crystals with diameters ranging from 0.05 to 1.3 mm, Aver’yanov et al. (1985) designed suitable snow layer structures according to different aircraft types and paid attention to the periodicity and type of snow layer treatment to ensure aviation safety and efficiency [65].

There are many research experiments on snow compression, whether calculating the snow load of buildings, designing and manufacturing vehicles that can run on snow, or using snow as building materials for roads, airstrips, and building foundations in cold areas [55]; it is necessary to understand the mechanical properties of local snow through snow compression experiments, which is an indispensable part of snow research in cold areas.

#### 2.2.3. Snow Failure in Mixed Failure Type

Mixed-mode snow disruption is ordinary, whether as minor as the compression and shear mix interaction between snow tires, skis, and snow or as large as the stretching and shear mix interaction within an avalanche. The experimental research on the damage of the snow mass by a mixed-mode load mainly focuses on the area where the weak snow layer breaks when the snow collapses and avalanches are released.

In the study of snow avalanches on dry slabs, Heierli et al. (2008) found that the snow block cracks were propagated and released along the weak layer under the mixed-mode loading of shear and compression [66]. In addition, the angle at which the avalanche is released depends mainly on the extent of the friction between the crack surfaces that have already spread at the time of the weak layer fracture [67]. Mede et al. (2018) observed three failure modes of snow blocks under the mixed-mode loading of different normal stresses with X-ray scanning microscopy and developed a discrete element numerical model to simulate a large-strain response of snow samples [68]. They thus gained a deeper understanding of the mechanical response of snow under mixed-mode loading. The Mohr–Coulomb principle is usually used to analyze the loading condition of the snow block under shear loading [69]. Reiweger et al. (2015) proposed a new mixed-mode shear compression failure criterion for a more comprehensive analysis of avalanche release [7]. However, due to the unique material properties of snow, the study of the internal changes during the collapse of snow blocks still needs to be completed. Mede et al. (2020) combined microstructure-based discrete element modeling and progressive micromechanical analysis to conduct a mathematical simulation of mixed-mode loading experiments but did not consider the viscosity effect of snow blocks and the characteristics of grain sintering [70]. On the other hand, Mulak et al. (2019) focused on the equilibrium relationship between bond breakage and bond formation among internal snow particles under mixed-mode loading [71].

By studying the mixed failure mode of snow blocks, we can better understand the interaction mechanism of the different failure types of snow blocks and the degree of influence between each other, which is of great significance for engineering applications.

**Figure 2:**
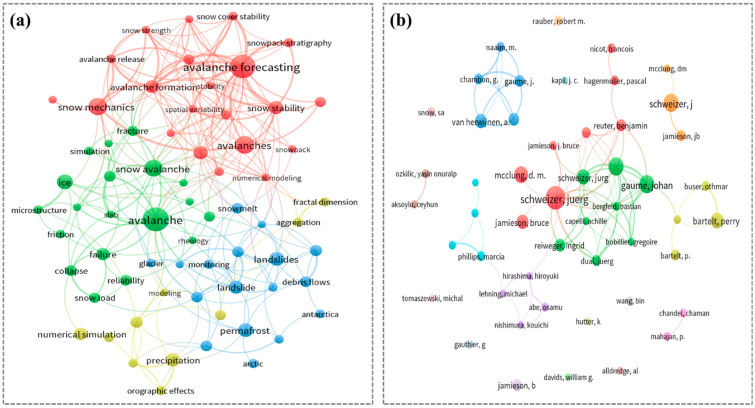
(**a**) Snow failure types’ keywords. (**b**) Authors heat picture in Snow failure research.

Upon reviewing the aforementioned literature, it becomes evident that the experimental study of snow failure plays a crucial role in understanding the characteristics of snow and the mechanism of snow failure. We used software VOS viewer 1.6.19 to produce Figure 2 which presents some keywords about snow failure and some authors who have conducted much research in this field. In order to better understand snow failure experiments, Table 2 summarizes studies, differentiating between the fundamental experiment information on snow failure.

## 3. Physical Description Models and Numerical Simulation Analysis

This chapter provides an overview of the research about numerical simulation. In Section 3.1, the numerical analysis model of snow failure is expanded from the analysis models, which study the microstructure changes in the process of snow failure at small and medium scales, to the numerical simulation model and method applied to large-scale avalanche analysis. Section 3.2 summarizes the approaches and methods of acquiring 3D snow models in recent years, and the formation and prevention of avalanches are summarized based on the numerical modeling of avalanches.

### 3.1. Physical Description Models

#### 3.1.1. Microscopic Analysis Models

Microscopic analysis models provide structured insight into snow failure simulation research, which is fundamental for a profound understanding of snow behavior, particularly within the challenging environmental conditions of Antarctica. The models depicted, including the finite element method (FEM), discrete element model (DEM), and fiber bundle model (FBM), collectively constitute a crucial framework for researchers and practitioners to assess snow stability, predict avalanche risks, and formulate effective mitigation strategies in polar regions.

**Figure 3:**
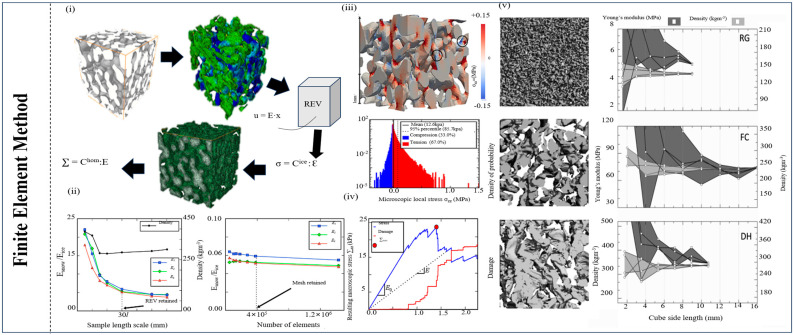
**Summary of FEM simulation method** [87,89,90]. (**i**) FEM simulation flow chart and related formulate. (**ii**) Convergence analysis of Young’s modulus and density of snow sample. (**iii**) Three-dimensional representation for 3 × 3 × 3 mm^3^ snow block, circled areas show two bonds undergoing bending deformation and histogram of stress distribution. (**iv**) Macroscopic stress and percentage of damage as function of macroscopic strain. (**v**) RVE calculations for Young’s modulus and density (right y-axis, light-grey area) vs. cube side length.

The finite element method (FEM) typically treats the simulated material as a continuous entity and employs a constitutive model to capture its response under various loading conditions, which is one of the popular numerical analysis methods in continuum mechanics and mechanics of solids. It is commonly combined with X-ray microcomputer tomography technology to perform the three-dimensional modeling of snow blocks, followed by a finite element numerical simulation. Figure 3 shows the typical flow chart of the FEM method. Kochle et al. (2014) calculated Young’s modulus and Poisson’s ratio through finite element simulation based on X-ray tomography [86]. However, none of the parameters except Young’s modulus could consistently explain the structural changes [86]. Gerling et al. (2017) compared the elastic modulus measurement in snow by the finite element method with acoustic propagation (AC) and high-resolution digital cone penetration measurement (SMP) [87]. The moduli (10–340 MPa) obtained from acoustic and finite element methods are found to be very consistent over the entire density range (170–370 kg/m^3^) [88]. Due to snow’s many pores and sintering behavior, finite element methods specially designed for continuum materials are hindered. Hagenmuller et al. (2014) inputted the viscoelastic and bond fracture behavior of ice crystals into the model [88]. The pseudoplastic yield curve is formed when the simulation involves the nonlocalized failure of ice lattice bonds, whether before or after reaching the peak stress. It is applied to more complex snow layer structures [89]. Chandel et al. (2014) simulated the strain-softening behavior of snow by using an ice elastoplastic constitutive model based on damage [89]. They studied the accuracy of mechanical properties obtained through numerical simulation in combination with an unconfined constant variability experiment [89]. In conclusion, the FEM has a wide range of applications in snow mechanics, including the transfer of ground loads under snow cover, the mechanical response of avalanches, and the mechanical optimization of sleds and snowboards. This method can better understand snow’s deformation and stress distribution, as well as guide related engineering design and safety assessment. At the same time, researchers continue to improve and optimize FEM models for the pore structure and complex mechanism of snow.

**Figure 4:**
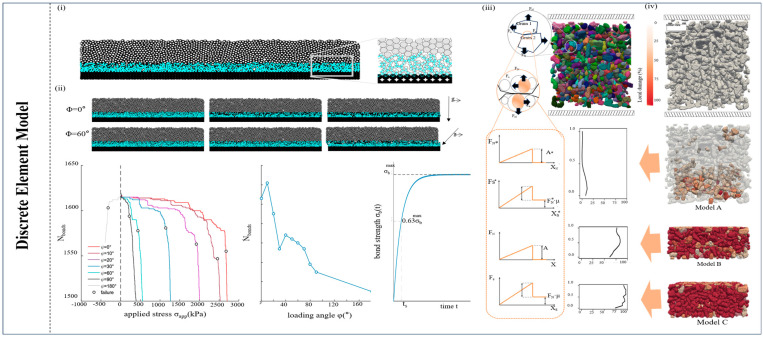
**A summary of DEM numerical simulation** [68,70,71]. (**i**) The simulated weak snow layer (blue)—slab (gray) system and the magnified image. (**ii**) Different states of snow under pressure simulation, cohesive bonds as a function of the applied stress, and the number of broken bonds at failure as a function of the loading angle. (**iii**) The snow sample was modeled in the DEM, along with boundary conditions, and applied to the load. Individual grains are marked with varying colors for clarity. (**iv**) Three snapshots of the distribution of the local damage at different times and the final vertical profile of the damage.

The discrete element model (DEM) is another microscopic analysis model that adopts a particle-based approach to simulate snow behavior. Figure 4 presents some typical simulation figures and relations of parameters in the DEM. This model represents snow as a collection of discrete particles or elements that interact with each other through contact forces, corresponding to individual snow grains or particles, which is able to describe micromechanics at grain contacts explicitly and identify the important processes that control snow deformation [91]. In the deformation of snow, crystal adhesion and structure destruction occur inside the snow, affecting its mechanical properties. Hagenmuller et al. (2015) used the DEM approach to simulate dry snow deformation at a high loading rate, showing the result that the mechanical behavior of snow is mostly controlled by density [92]. Furthermore, Theile et al. (2020) conducted a numerical analysis of large strain processes in snow compression and shear experiments [93]. They limited the model to high strain rate deformations where creep deformation can be neglected, which successfully captured the breaking behavior of snow blocks, the difference in different density snow, and the densification behavior of low-density snow [93]. However, the consideration of sintering behavior and temperature factors is missing. [93]. Kabore et al. (2021) regarded the microstructure of snow as multi-crystalline ice grains [94]. They proposed a discrete element model that considered the factors of grain collision, deformation, fracture, and bond growth due to the creep of ice and temperature to improve the damage between snow crystals and the energy dissipation mechanism of grains [94]. The DEM proves particularly invaluable for simulating the dynamic behavior of snow grains and their role in snow failure mechanisms. It adeptly captures the intricate interplay between particles, including collisions, friction, and cohesion, which are fundamental to understanding how snow behaves under various conditions. Huo et al. (2023) used the three-dimensional discrete element method to study the deformation behavior and microscopic mechanism changes in snow samples with different densities in the process of compression, fully demonstrating the behavior of snow samples in the process of deposition, compaction, and sintering in combination with experiments, and discussed the fracture of intergranular and intragranular bonds in detail [95].

The DEM allows researchers to explore granular flow phenomena, which are critical in comprehending snow failure processes such as avalanches and snow slides. By simulating the behavior of numerous particles within a snowpack, the DEM provides valuable data on the initiation and propagation of snow failures, as well as the contributing factors. This micro-scale analysis is essential for evaluating the stability of snow-covered slopes in Antarctica, where the interaction between individual snow grains and their response to external forces carries significant implications for safety and infrastructure management. The DEM contributes substantially to the development of more accurate and predictive models of snow failure in polar environments, enhancing our ability to anticipate and mitigate the risks posed by snow-related hazards in this challenging region [73,96,97].

**Figure 5:**
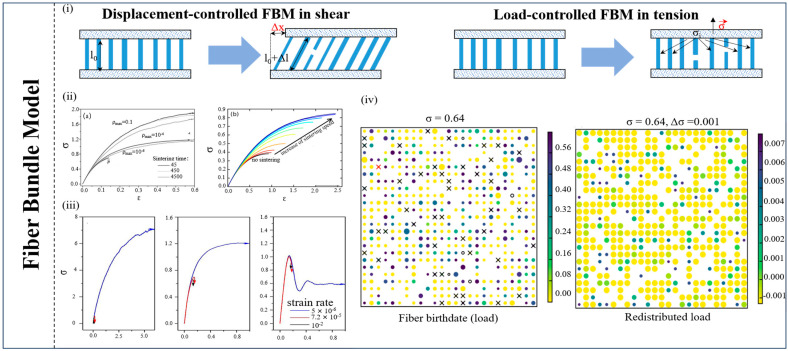
**A summary of the FBM numerical simulation** [98,99,100]. (**i**) Two models of theories of the FBM. (**ii**) Stress–strain relations of two FBM models, (a) comes from the first model in (**i**), (b) comes from the second model in (**ii**), different colors mean different sintering load. (**iii**) The ductile-to-brittle transition of the displacement-controlled FBM model. (**iv**) A snapshot of the FBM for a single load step in the form of 2D maps of the fiber bundle illustrating the sintering and load relaxation processes.

The fiber bundle model (FBM) was originally a statistical fracture model [74]. As shown in Figure 5, it was first used by Reiweger et al. (2009) as a microscopic analysis model of snow to study the influence of microscopic structure parameters on the overall mechanical response of the snow layer by simulating the deformation and failure of the snow layer [98]. Within the FBM, snow is conceptualized as a network of interconnected fibers or elements, each endowed with its inherent strength, representing individual snow grains or particles. The displacement control model used by Reiweger et al. (2009) in the FBM simulation presented the mechanical properties of the ductile to brittle transition of snow and introduced the concept of fiber fracture sintering [98]. Capelli et al. (2018) went a step further and adopted a load-controlled fiber bundle model, which assumed that the fibers gained full strength immediately after sintering, reducing the influence of time variables and reducing the complexity of the model [99]. Combined with the function of load relaxation, it is more suitable for the actual process of snow layer failure [99]. In addition, the FBM, with this model, can also verify the acoustic emission (AE) characteristics in the snow failure experiment and the relationship between load rate and the stress–strain curve [100]. This model offers a strong framework for comprehending the initiation of snow failure at the micro-scale, allowing researchers to investigate the way that variations in the strength of individual snow grains impact the overall stability of snow masses. Through simulations of the interactions between these fibers, the FBM provides valuable insights into how snow structures evolve under external forces like temperature changes, mechanical loading, or stress from additional snow accumulation. Such simulations facilitate the assessment of factors such as snowpack cohesion, grain size distribution, and the influence of various environmental conditions on snow stability, thereby playing a critical role in predicting and mitigating avalanche risks and other snow-related hazards in Antarctica, where the heavy snowfall, low temperatures, and volatile weather conditions pose unique challenges.

Other simulation models used in snow failure, such as the open-cell foam model (OCM), are employed to decipher snow behavior by breaking it down into smaller, interconnected components [101]. In this approach, snow is envisioned as a collection of interconnected open cells, mirroring the structure of a foam material, with each cell representing a discrete unit of snow within the larger mass. OCM simulations can be harnessed to investigate phenomena such as the metamorphism of snow crystals, which can trigger changes in snowpack stability over time [102]. In addition, Meyer et al. (2017) used a continuum model to study the percolation process inside snow that includes heat conduction, meltwater percolation, and refreezing [103]. On the other hand, a local lattice model (LLM) was proposed to describe the growth process of snow crystals [104]. In conclusion, we present Figure 3, Figure 4 and Figure 5 to summarize the main microscopic numerical simulation models.

#### 3.1.2. Macroscopic Analysis Models

**Figure 6:**
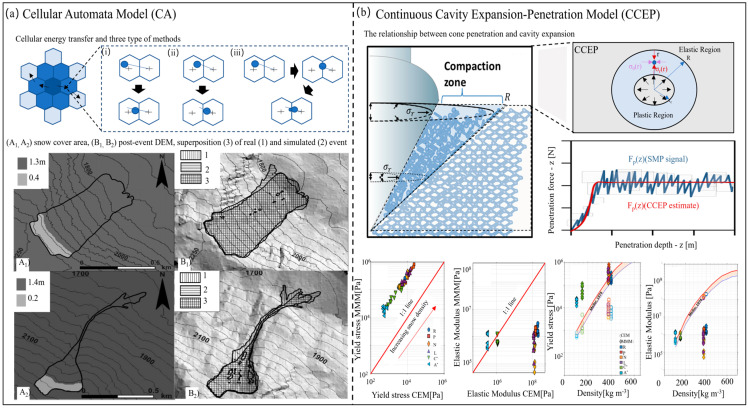
**Macroscopic analysis models in avalanche research.** (**a**) The cellular automata model simulates the flow of dense avalanches, (i) All the cylinder remains in the cell: the flow is only internal (ii) All the cylinder leaves the cell: the flow is only external. (iii) Part of the cylinder crosses the cell: there are both internal and external flows [111]. (**b**) The relationship between cone penetration and cavity expansion in the CCEP model and a comparison between the bulk snow yield stress and the bulk snow effective elastic modulus obtained from the micromechanical model (MMM) and the cavity-expansion penetration model [112].

**Figure 7:**
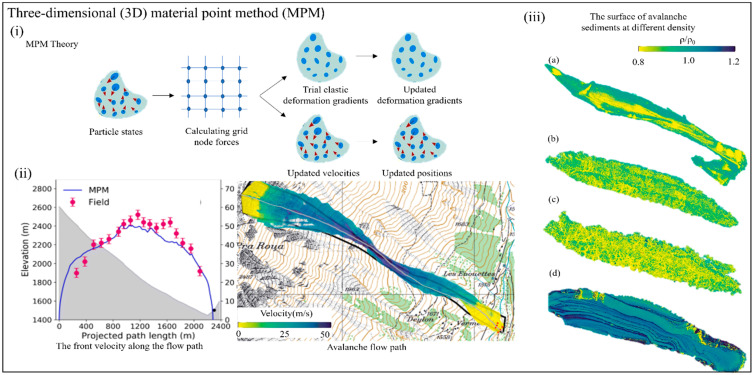
**Macroscopic analysis model of the 3D MPM in avalanche research.** (**i**) Overview of the MPM algorithm. (**ii**) The three-dimensional (3D) material point method (MPM) is used to explore different avalanches on complex real terrain, and the flow characteristics of avalanches from release to deposition are comprehensively characterized. (**iii**) Four typical distribution of density ratio in avalanche [110].

Figure 6 and Figure 7 succinctly generalize a scholarly exposition of macroscopic analysis models, delineating their critical role in comprehending avalanche dynamics and safeguarding human lives and infrastructure. Particular emphasis is on the cellular automata (CA) model which provides good simulation effects on the flow path, snow cover, and avalanche erosion depth of a dense avalanche. The figure also shows the continuous cavity-expansion penetration (CCEP) model’s simplicity, versatility, and data-driven simulations, as well as the three-dimensional (3D) material point method (MPM) for exploring the different states of avalanches on complex actual terrain, which can comprehensively characterize the flow characteristics of avalanches from release to deposition. In these harsh environments, where avalanches can pose severe threats, macroscopic analysis models emerge as indispensable tools, revealing the intricate interplay of forces and variables governing snow avalanche dynamics.

The cellular automata (CA) model is suitable for simulating complex discrete dynamics by using simple rules to define the interactions between adjacent units of the discretized study area [105]. In a river basin surrounded by mountains, cellular automata (CA) models can simulate the dynamics of the daily spatial distribution of the snow cover area (SCA) and generate potential climate change scenarios to calibrate and verify the simulation of snow cover by the CA model [106], which plays a vital role in water resource management in alpine systems. As shown in Figure 6a, researchers used the VALANCA model to back-analyze two avalanches.

In addition, the continuous cavity-expansion penetration (CCEP) model assumes a prominent role as well, earning recognition among researchers and practitioners in the specialized field of avalanche science. This section delves deeply into the multifaceted significance of macroscopic analysis models; we discussed the CCEP model that is operated within the unique context of Antarctic snow failure mechanics [107]. With its inherent simplicity, the CCEP model simplifies the multifaceted behavior of snowpacks into a handful of critical variables, presenting it accessibly to a broader audience of researchers and practitioners [108]. Figure 6b simply presents the CCEP theory, which versatility is evident in its applicability across a broad spectrum of avalanche scenarios, encompassing dry and wet avalanches, slab avalanches, and even powder snow avalanches. Moreover, the model’s reality effect is exemplified by its comprehensive consideration of factors such as snowpack density, slope angle, and weak layer properties, enabling it to generate simulations that faithfully mirror the dynamics of real-world avalanches [109]. Crucially, the utility of the CCEP model extends far beyond theoretical speculation. Researchers engage in meticulous analyses fueled by data on snowpack properties, terrain morphology, and historical avalanches events specific to the Antarctic context. Armed with this data, they harness the predictive capabilities of the CCEP model to simulate avalanche behavior across diverse scenarios. In order to gain a deeper understanding of the various flow behaviors of avalanches, a three-dimensional material point method can be used to identify the brittle and ductile fractures of the slab produced in different simulated avalanches. Analyzing the local snow density changes during the snow flow shows that the formation of snow particles requires the appropriate combination of snow fragmentation and snow compaction [110].

Figure 7 shows the principle of the material point method (MPM) and the application of avalanche modeling; researchers used the topographic data from Vallée de la Sionne test site in Switzerland, characterizing the different textures of avalanche sediments, including smooth surfaces, rough surfaces with snow grains, and surfaces showing the compacted shear surfaces that are often present in wet snow avalanche sediment studies.

Macroscopic analysis models serve as the essential mitigation strategies of avalanche research. They help decipher the complex life cycle of avalanches, encompassing initiation, propagation, and runout phases. By distilling the intricacies of snowpack properties, terrain morphology, and external triggers into manageable parameters, these models provide a simplified and highly accurate representation of avalanche dynamics. This understanding forms the bedrock upon which effective avalanche mitigation strategies are built, thereby enhancing public safety and fortifying vital infrastructure in regions where the risks are exceptionally high, such as the Antarctic. These simulations offer invaluable insights into the mechanisms of avalanche initiation, the fluid dynamics of avalanche flow, and the patterns of avalanche runout. The practical applications of macroscopic analysis models extend their reach into multiple domains. Avalanche forecasters utilize these models to issue timely warnings and advisories, preserving human lives and safeguarding property in avalanche-prone regions. For engineers and architects, the insights garnered from simulation results are crucial in the design and construction of avalanche-resistant infrastructure, ensuring the resilience of critical lifelines. In the realm of emergency response, agencies responsible for managing disasters find these models invaluable for preparing for avalanche-related crises effectively [113].

### 3.2. Numerical Simulation Methods

#### 3.2.1. Three-Dimensional Solid Modeling of Snow

The three-dimensional solid modeling of snow represents a fundamental and indispensable approach for comprehensively unraveling the intricate structural and physicochemical attributes of snowpacks, especially at the micro-scale level. This is particularly pertinent across a spectrum of scientific disciplines, prominently including climate science, where a precise grasp of snow properties impacts climate models, avalanche prediction, wherein understanding snowpack stability is paramount for safety, and winter equipment design, which relies on snow’s mechanical characteristics for optimizing performance [114]. In this scholarly exposition, we shall carefully expound upon three vital methodologies employed in the creation of 3D solid models of snow, namely continuous sectioning, X-ray computed microtomography (micro-CT), and a simulation of the microcomputer tomography (μCT). Continuous sectioning entails the physical division of snow samples into slender slices, frequently executed through a meticulously calibrated microtome. Subsequently, the intricate and highly magnified images of these sections are meticulously acquired, often employing advanced optical or scanning electron microscopy techniques. This method is carefully planned through a series of clearly defined stages, starting with the preparation of snow samples to reduce structural perturbations [97,115,116,117,118], as well as the significance of X-ray computed microtomography (micro-CT) in elucidating snow’s intricate 3D microstructure. The study of snow mechanics and avalanche behavior is paramount for ensuring the safety of both human populations and critical infrastructure in regions prone to snow avalanches, such as the vast and remote Antarctic.

These models play a prominent role in environmental conservation by aiding in the mitigation of the environmental impact of avalanches in the fragile ecosystems of Antarctica. Concurrently, X-ray computed microtomography (micro-CT), an instrumental non-destructive imaging technique, assumes a vital role in the comprehensive visualization of the internal structural attributes inherent within snow samples. The method unfolds through a well-structured sequence of operations. Preliminarily, the requisite snow samples are prepared to ensure their alignment within the specialized confines of a micro-CT scanner. Subsequently, the micro-CT scanner undergoes a calibrated rotation around the snow sample while systematically collecting a multitude of X-ray projections. These projections are subsequently used in the creation of a volumetric 3D dataset that encapsulates the intrinsic internal structure of the snow sample. The critical phase of image reconstruction is executed through the deployment of advanced algorithms, which amalgamate the multitude of X-ray projections into a coherent and highly resolved 3D image [96]. This scanned image serves as a window into the intricate internal structure of the snow sample, representing a non-destructive and precise avenue for visualizing snow’s 3D microstructure. In light transmission in analytical and modeling contexts of snow crystals, the application of ray tracing methods assumes prominence in simulating the imaging process employed in microcomputer tomography (μCT). This intricate technique necessitates the creation of a virtual 3D model that truly mirrors the snow sample. Subsequently, it entails the meticulous simulation of the interactions between X-rays and the virtual snow structure. This simulation generated images that meticulously mimic those acquired through a tangible μCT scanner. The simulated μCT images, thus generated, are subject to rigorous data analysis, similar to their real-world counterparts, permitting the extraction of invaluable insights into the microstructural attributes of the snow sample under investigation. Ray tracing models in this context serve as a powerful tool for elucidating the nuanced effects of X-ray interactions on image quality, furthering our understanding of the optimization parameters inherent to μCT scanning [119].

**Figure 8:**
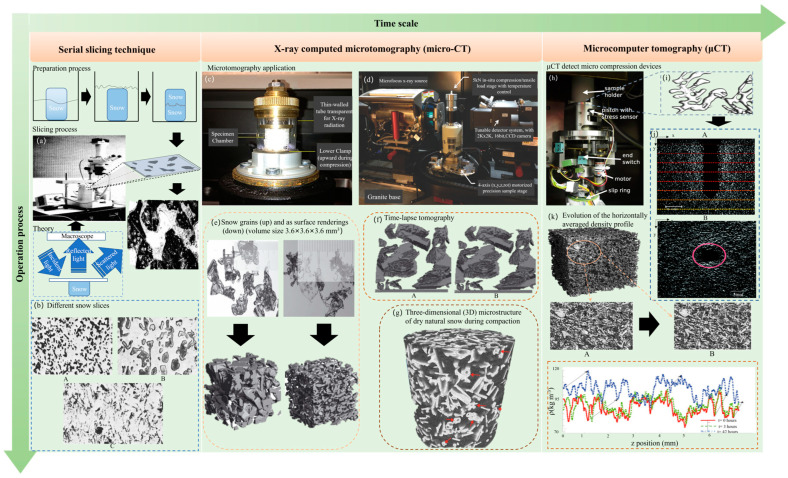
**A summary of the three-dimensional solid modeling of snow.** (**a**) Continuous sections and continuous micrographs are taken [122]. (**b**) Examples of snow sections [123]; image (A) is the partially metamorphosed snow of mid-density (−200 kg/m^3^), image (B) is the snow metamorphosed for 6 months at a weak temperature gradient (−1 °C/m), and image (C) is the snow metamorphosed for 100 h in an intense temperature gradient (>1000 °C/m). (**c**) The SkyScan material test bench for the micro-CT system for scanning snow blocks [124]. (**d**) The experimental arrangement of the micro-CT system [125]. (**e**) Typical snow types presented as snow grains (left) and as surface renderings (right) [126]. (**f**) Two images separated by 2 days from a time-lapse movie [126], image (A) at time 577 h, image (B) at time 625 h. (**g**) Three-dimensional images of the distribution of snow grains [125]. (**h**) μCT detect micro compression devices [127]. (**i**) The snow grains are imaged under the microscope [128]. (**j**) An overview of μCT image analysis [129]; (A) is the picture of the snow vertical transect μCT and (B) is the picture of snow horizontal transect μCT. (**k**) An X-ray microtomography analysis of the isothermal densification of new snow under external mechanical stress [130].

Snow samples are invariably ensconced in a suitable medium to ensure the preservation of their native morphology. The ensuing phase involves the precise sectioning of the embedded snow sample into razor-thin sections via a microtome or a cutting apparatus. These sections span a range from mere micrometers to several millimeters in thickness. Each section is carefully scrutinized under the discerning gaze of a high-powered microscope, eventually capturing high-resolution images that serve as the fundamental building blocks for the subsequent 3D reconstruction process. This meticulous reconstruction achieved through specialized software entails the precise alignment and stacking of the 2D images originating from the diverse sections. The prominence of this labor-intensive endeavor is the emergence of an exact 3D representation that affords intricate insights into the shapes and arrangements of individual snow crystals, thereby serving as a crucial tool for microstructural analysis [120]. Figure 8 provides a concise visual summary of the three-dimensional solid modeling of snow, illustrating the critical stages from data acquisition and modeling techniques to numerical simulations, applications, and the field’s advancements. This figure offers a clear overview of the comprehensive process involved in understanding and utilizing three-dimensional solid modeling in snow research [121].

#### 3.2.2. Avalanche Behavior in Snow Failure and Simulation in Practical Application

**Figure 9:**
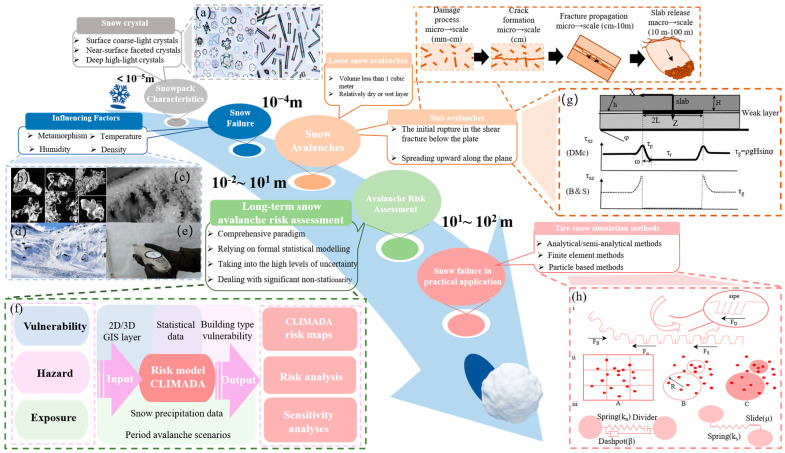
**A summary of the avalanche formation mechanism and release process.** (**a**) Different types of snow crystals [38]. (**b**) Photomicrographs of metamorphic crystals frequently observed in snowpacks [139]. (**c**) Snowpack crystals [140]. (**d**) Wet plate avalanche [141]. (**e**) The measurements of snow’s mechanical properties [142]. (**f**) A schematic illustration of the applied IPCC risk concept, which is used to create risk maps and perform risk analyses [143]. (**g**) Snow slab release models with pre-existing weakness [131]. (**h**) Three tire–snow simulation methods. (i) F_S_ is the shear force of snow in void (space between tread blocks), F is the frictional force between tire and snow, F_µ_ is the digging force (edge effect generated by sipes and blocks), and F_B_ is the braking force. (ii) Computing a continuous density field from a collection of point mass particles. (A) is the particle-based method, (B) is the way to calculate based on the size of the sample volume, and (C) is the SPH method. (iii) The DEM contact model between two contacting particles, (left) normal contact model, (right) tangential contact model [144].

Figure 9 presents a concise visual summary of the avalanche formation mechanism and release process, highlighting how different factors influence the assessment of avalanche risks and the development of mitigation strategies, ultimately ensuring safety in snow-prone regions. In this section, we embark on an exploration of snow failure simulations, delving into a myriad of scenarios that encompass the complex mechanisms governing avalanches, the intricate interactions between tires and snow in the Antarctic environment, and the profound implications of wind-induced snow erosion on structures.

Our academic journey commences with meticulous scrutiny of Antarctic snow drifts, where we undertake a multidisciplinary analysis that delves deep into the snow accumulation dynamics and the unique geographical features of this polar region. This meticulous research affords us a comprehensive understanding of the factors contributing to snow drift formation in the frigid and inhospitable Antarctic landscape, advancing our knowledge of climatology, geography, and snow science. In the field of snow mechanics, avalanches are one of the important research topics related to snow failure. Avalanche release starts with the compression or shear deformation of the snow block and is affected by external temperature, humidity, wind erosion, metamorphism, and other factors, resulting in cracks. When the cracks reach a certain threshold, they expand to the surrounding snow block, eventually leading to an avalanche. It is difficult to predict the occurrence time and size of a single avalanche, but avalanche risk assessment and corresponding mitigation strategies can be made based on the avalanche history, occurrence probability, and other information about the area [131].

Subsequently, our academic discourse transitions to the realm of tire–snow interactions within the Antarctic context. Relative analysis incorporates a consideration of various critical factors, including tire design, surface properties, and the prevailing environmental conditions, to unravel the intricacies that govern vehicular mobility and safety in these extreme polar conditions. Numerical simulation is a more efficient and convenient method for snow-tire research due to the time-consuming field testing. The analytical/semi-analytical method was established by Nakajima (2003), which was used to estimate the longitudinal traction of a tire in snow [132]; Mundl et al. (1997) used the Lagrange method in the finite element method (FEM) to establish an elastoplastic material model of snow, enabling a comprehensive investigation into the interaction between elastic tread blocks and inelastic snow surfaces [133]. Particle-based methods have become increasingly popular in recent years; as shown in Figure 9h, El-Sayegh et al. (2019) used the smooth particle hydrodynamics (SPH) method to model snow and calculate the motion resistance coefficient of the truck tire–snow interaction, and the effects of vertical load, tire longitudinal speed, and snow depth on the resistance coefficient were investigated [134]. On the other hand, Xu et al. (2020) applied the FEM and DEM to study the interaction between off-road tires and granular terrain [86]. Then, they used triaxial compression experiments to verify the relationship between tire traction performance parameters and the slip rate [86]. Through this scholarly investigation, we contribute valuable insights to transportation engineering, materials science, and polar research, facilitating the development of cutting-edge solutions for navigating challenging snow-covered terrains.

In addition, our scholarly study includes almost every aspect of avalanche science, encompassing the multifaceted factors that contribute to avalanche initiation, propagation, and ultimately, the formulation of effective mitigation strategies [135]. These insights are of paramount significance, as they hold the potential to safeguard lives and protect critical infrastructure in avalanche-prone regions, making substantial contributions to the fields of geophysics, geology, and avalanche engineering. Finally, we navigate through the formidable arena of wind-driven snow erosion, exploring how winds sculpt and reshape snow surfaces. Our academic investigation extends to the evaluation of structural risks posed to buildings and installations, and we contemplate innovative engineering and architectural design solutions that can withstand the forces of nature in snow-prone environments. By doing so, we significantly contribute to the domains of civil engineering, architectural design, and snow science, advancing the knowledge and practices associated with mitigating the challenges posed by wind-driven snow erosion [136].

Avalanches are very complex natural disasters. Researchers have conducted extensive and in-depth research on snow’s physical and mechanical properties to better understand the snow layer’s stability, the evolution of snow structure, and mechanical behavior. Various models and methods have been developed, such as models based on snowfall and numerical simulations of terrain analysis. Moreover, field observations and mathematical–statistical models have been combined to provide a certain degree of avalanche prediction and early warning. However, it is still impossible to predict a single avalanche’s flow direction and trigger time. Because of avalanches’ highly nonlinear and multi-scale characteristics, it is still difficult to establish a very accurate model and digital simulation [137]. In the future, avalanche research will have multi-disciplinary cooperation and cross-disciplinary trends; consider regional climate change, terrain characteristics, and other data; and combine machine learning to develop more suitable and accurate numerical simulation models [138].

## 4. Applications of Snow Failure Mechanics in Antarctic Infrastructures

The engineering challenges posed by Antarctica’s unique climatic conditions necessitate advanced solutions for ensuring the regular functionality of crucial infrastructure, particularly pavement construction and maintenance. Snow can also be regarded as a collection of fine gravel, so constructing a snow runway is similar to traditional flexible or rigid roads, which requires the consideration of stress distribution and load dispersion [145]. In addition, due to snow’s sintering and recrystallization properties, snow roads can easily be affected by temperature and density, so it is necessary to select appropriate seasonal weather during construction and test for snow density and strength during road compaction. Therefore, we require an analytical method for the characterization of snow based on temperature history and density [146]. The U.S. Army Corps of Engineers conducted a comprehensive test of snow runways in Antarctica in the 1960s, observing the change in the snow intensity of the snow roads when the aircraft landed to determine the appropriate snow intensity parameters for construction [145]. Russell-Head et al. (1984) tested the snow compaction and hardness of the snow track in the area of Law Dome near Casey, Antarctica [147]. They found that the California Bearing Ratio (CBR) strength of compacted snow was more significantly affected by density than temperature [147]. In addition, snow density during compaction is more straightforward to increase at higher temperatures [147]. Barber et al. (1989) added wood as a binder material to the snow runways and pavement at the South Pole stations McMurdo and Amundsen [148]. They found that satisfactory strength was obtained for supporting wheeled aircraft at depths of 20 cm below the snow ground at both stations [148]. In conclusion, constructing a snow runway involves several steps, including breaking up the snow, compressing it, leveling it, and then sintering [146]. In the process of construction, snow plows, ice edge breakers, and graders are first used to clean and standard the snow runway, then snow blocks are crushed and laid on the runway by snow powder machines, and then the runway is compacted with D8 bulldozers and Sheep hoof rollers [149]. The whole process is repeated several times according to the actual situation. In addition, the timing is crucial for snow runway construction. Processing and compaction should be carried out during periods of warm weather, allowing for sintering to occur during colder temperatures. A comprehensive understanding of snow failure mechanics assumes importance in this context. Fundamental aspects, such as pavement design and thickness, leverage snow mechanics insights to ascertain optimal thickness specifications capable of withstanding substantial snow loads without deformation or failure.

The judicious selection of pavement materials, informed by snow mechanics analysis, becomes instrumental in withstanding the harsh environmental factors, including extreme temperature fluctuations and the abrasive forces exerted by snow and ice. The mathematical model of the snow load distribution on pavement is developed based on the snow mechanics principle, guiding the design of pavements that can resist deformation and damage caused by snow accumulation. For example, the snow road at McMurdo Station in Antarctica is a necessary transport route for people and supplies. It is susceptible to warm temperatures in the summer, thus changing the intensity of the snow road. Shoop et al. (2013) conducted a series of tests on four types of vehicles, such as which tires cause less damage to the surface [150]. Thus, the impact of different types of vehicles on snow roads in various weather and road conditions is assessed and guides distinguishing between which vehicles have a small impact and which have the most significant impact [150]. This knowledge guides vehicle and equipment usage to prevent damage to pavements. In regions where permafrost lurks beneath pavements, snow failure mechanics facilitate the design of pavements to ensure safe vehicular and equipment operations in Antarctica. Furthermore, an assessment of thermal performance in various pavement types and designs, aided by snow mechanics analysis, informs the choice of insulation and heating systems to thwart frost heaving and maintain pavement integrity [151]. Efficient snow removal is critical for pavement safety, and understanding snow mechanics informs the design and selection of snow removal equipment and strategies, minimizing pavement surface damage. Given Antarctica’s ecological sensitivity, environmental considerations are important. Snow mechanics studies contribute to developing sustainable pavement infrastructures that exert minimal ecological impacts on the region’s delicate ecosystems. Ensuring access to research stations and critical facilities is indispensable for scientific operations in Antarctica.

In summary, the applications of snow failure mechanics in Antarctic pavement infrastructures constitute an indispensable component in the booming construction, maintenance, and sustainability of essential transportation networks. By utilizing the principle of snow mechanics, engineers can devise pavements that are capable of enduring the extreme conditions of the Antarctic continent, thereby facilitating scientific research and logistical support while minimizing environmental consequences. These pioneering solutions stand as indispensable enablers for the exploration and comprehension of one of the world’s most remote and challenging environments [152].

## 5. Conclusions

In this review, we began by exploring the specific properties of snow, such as its porosity, the structure of snow crystals, and the sintering properties when bonded, and focused on the crucial factors that affect the properties of snow, including density, humidity, temperature, wind erosion, and metamorphism within the snow block. In the study of snow blocks, we discussed its mechanical properties through the snow failure experiment, including the failure process of snow blocks under compression, shear, tensile, and mixed modes, to obtain mechanical data, such as the elastic modulus, tensile strength, and shear strength, and understand the stress–strain relationship, stress–displacement relationship, ductility and brittleness deformation characteristics, and sintering characteristics. Furthermore, numerical simulation is a critical method to study snow failure behavior. We described the methods of acquiring 3D model data of snow blocks and their development process, including continuous section, X-ray microtomography, and tomography. Subsequently, numerical models commonly used at the microscopic scale, such as the fiber bundle model (FBM) and discrete element model (DEM), were introduced to gain an in-depth understanding of the failure behavior and mechanical properties of snow blocks under compression, shear, tensile, and mixing modes. At the macro-scale, snow failure manifests as avalanches triggered by weak-layer snow slab fractures. We described numerical simulation methods such as the continuous cavity-expansion penetration model (CCEP), cellular automaton model (CA), and three-dimensional material point method (MPM) for avalanche risk warning and prevention measures. Then, we reviewed the formation mechanism, the release process of the avalanche, and the corresponding prevention mechanism. Lastly, we discussed the application of snow failure mechanics, such as the construction of the Antarctic snow runway and snow–tire interfacial relationship research. In conclusion, this review focused on the mechanical characteristics of snow, the destructive behavior of snow, and some applications of this kind of research, which has academic significance for the construction of snow structures, snow tire design, and avalanche warning and prevention in the future.

## Figures and Tables

**Table 1:** Snow sample processing parameters [26].

Snow Properties of Different Snow Samples				
No.	Density (kgm^−3^)	Grain Size (mm)	Snow Type	Image Sample
1–2	93–107	<0.5	New snow	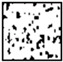 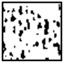
3	109	1	Nearly new snow	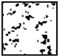
4	159	0.1–0.5	Slightly densified new snow	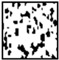
5	162		New snow with surface hoar	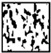
6	177	0.5–1	Nearly new snow	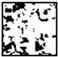 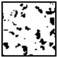
7	191			
8–9	231–244	0.1–0.5	Hard, densified snow	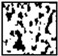 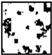
10–11	367–384	0.1–0.5	Hard snow	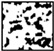 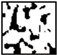
12–13	240–332	1	Coarse grains	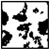 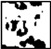
14	252	0.7	Densified snow	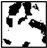
15	260	0.1–0.4	Strongly densified snow	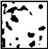
16	322	0.5–1	Hard slab	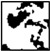
17	335		Rounded edges	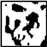
18–19	270–279	1–3	Depth hoar	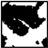 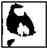
20	345		Depth hoar, rounded edges	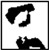

**Table 2:** Snow failure experiment and practical application.

**Experiment Context for Snow Failure**				
**Type**	**Parameter**	**Result Analysis**		**References**
Shear	Load speed Load pressure Temperature Snow type Relative Permittivity Time scale Young’s Modulus	Modulus of elasticity Tensile strength Shear strength Pore pressure Crack propagation Stress–strain relation Stress–displacement relation Ductile–brittle deformation Fragmentation Index (Fr. I)		[40,41,42,45,47,59,72],
Tension				[6,44,45,59],
Compression				[48,49,50,51,52,53,73,74],
Slope of repose				[75,76]
**Experiment Instruments for Snow Failure**				
**Instrument Name**	**Test Contexts**			**References**
Push–pull gauge	Snow hardness measurement		--	[77]
Ram/RSP			Snow density measurement	[78]
Snow micro-penetrator	Snow strength measurement			[79]
Density cutter device				[80]
Light-hammered Clegg impact tester			Compaction of snow layer	[81]
CTI snow compaction gauge				[82]
Vibrating device	Shear strength measurement of snow blocks		--	[83]
Bevameter rotating device			--	[40]
Force deformation-control shear device	Loading snow sample by gravitational force		--	[84]
Shear box	The mechanical characteristics of weak snow layers in avalanche release zones		--	[85]
